# Comprehensive Factors for Predicting the Complications of Diabetes Mellitus: A Systematic Review

**DOI:** 10.2174/0115733998271863231116062601

**Published:** 2024-01-04

**Authors:** Madurapperumage Anuradha Erandathi, William Yu Chung Wang, Michael Mayo, Ching-Chi Lee

**Affiliations:** 1 University of Waikato, Hamilton, New Zealand;; 2 National Chen Kung University Hospital, Tainan, Taiwan

**Keywords:** Diabetes mellitus, risk factors, machine learning, complications of diabetes, cholesterol, triglyceride, BMI

## Abstract

**Background:**

This article focuses on extracting a standard feature set for predicting the complications of diabetes mellitus by systematically reviewing the literature. It is conducted and reported by following the guidelines of PRISMA, a well-known systematic review and meta-analysis method. The research articles included in this study are extracted using the search engine “Web of Science” over eight years. The most common complications of diabetes, diabetic neuropathy, retinopathy, nephropathy, and cardiovascular diseases are considered in the study.

**Methods:**

The features used to predict the complications are identified and categorised by scrutinising the standards of electronic health records.

**Results:**

Overall, 102 research articles have been reviewed, resulting in 59 frequent features being identified. Nineteen attributes are recognised as a standard in all four considered complications, which are age, gender, ethnicity, weight, height, BMI, smoking history, HbA1c, SBP, eGFR, DBP, HDL, LDL, total cholesterol, triglyceride, use of insulin, duration of diabetes, family history of CVD, and diabetes. The existence of a well-accepted and updated feature set for health analytics models to predict the complications of diabetes mellitus is a vital and contemporary requirement. A widely accepted feature set is beneficial for benchmarking the risk factors of complications of diabetes.

**Conclusion:**

This study is a thorough literature review to provide a clear state of the art for academicians, clinicians, and other stakeholders regarding the risk factors and their importance.

## 1 INTRODUCTION

The high prevalence rate of diabetes mellitus (DM) and its complications is leading to a severe global health burden [1, 2]. Due to the high demand for expert knowledge, the soaring expenses in the healthcare sector, and the high cost of diagnostic tests and equipment, developing health decision-making systems through machine learning (ML) techniques has gained much attention in assisting disease diagnosis and prediction [3-5]. Classification, time-series analysis, clustering, and predictive analysis are well-known ML techniques in designing systems in the healthcare context to accomplish purposes, such as categorising the dataset, analysing and finding patterns over a period, and forecasting the future. Although several acute and chronic complications are consequences of DM, diabetic retinopathy (DR), diabetic neuropathy (DNeu), diabetic nephropathy (DNep), and cardiovascular diseases (CVD) can be considered the most frequent and severe complications [6]. The potential repercussions and irreversibility of the complications mentioned above of diabetes mellitus (CoDM) lead to the development of risk prediction/scoring systems.

Predicting diabetes and its complications using ML techniques is evolving as a prominent research area due to their potential health outcomes and ability in personalised medicine. The cost-effectiveness, utilisation of expert knowledge, and enhancement of health indices from ML-aided systems made them more popular in the digital healthcare industry. Feature selection is one of the vital steps that the model designer should perform while developing risk prediction/scoring models. However, predicting the risk of CoDM is challenging due to confounders and the existing disputability of a standard feature set [4, 7]. Therefore, this study aims to assist in the feature selection phase of model development by creating the most frequently used feature sets for each CoDM by conducting a systematic review according to a well-known scientific method, PRISMA.

Systematic reviews are commonly used for summarising research findings in health care [8]. Stakeholders in healthcare settings utilise periodic review articles to fulfil various purposes, such as academics seeking comprehensive literature overviews, clinicians relying on evidence-based guidelines, and doctors staying informed about the latest research developments. Furthermore, this can be considered a justification for building clinical practice guidelines. The justification of the requirement of further research in the related topic may be useful for granting agencies [9]. Primary care/ family physicians use systematic reviews as decision-making tools. The identified gaps in existing prediction models recognised beneficial or harmful medical interventions, summarised through systematic reviews, are valuable for clinicians, researchers, the public, and policymakers [8]. Furthermore, inaccurate and ineffective risk-scoring models result from the need for a standard set of risk factors in predicting CoDM [4, 7]. Therefore, extracting a standard set of features that can be used in multi-ethnic models is crucial. Moreover, electronic health records (EHR) are becoming an eminent research focus on health informatics [10], which can extract patient details effectively. Due to the divergence of national health information stored in different countries, considerable variations can be seen in EHR among countries. United States Core Data for Interoperability (USCDI) makes a consensus to achieve interoperability. The data types used in USCDI are demographics, diagnoses, problem lists, family history, allergies, immunisation, medications, procedures, lab orders/values, vital signs, reports, and utilisation. There are a further nine data types under emerging data types: Bio-sample data, genetic information, social data, patient-generated, community, geo-spatial, surveys, free text, and other data types [11]. Due to the popularity and effectiveness of EHR in health informatics, it is vital to focus on the most commonly available features in EHR when extracting features for creating prognosis or diagnosis models. This study adopts the categories of data types in EHR by considering the USCDI standardised method to categorise the features extracted from the literature. The research study focuses on extracting a frequently used feature set for predicting the selected set of complications of diabetes. A systematic review has been conducted to fulfil the aim of the research with the PRISMA standards.

This systematic review is conducted to answer the existing research gap to a considerable extent while fulfilling the objectives:

1. Determining the frequent risk factor lists used to predict DR, DNeu, DNep, and CVD.
2. Briefly describe the highly prioritised frequent factors.

The research method and materials are included in section 2, with a description of the process of article selection and feature extraction. The result and discussion sections include the extracted feature set and brief descriptions of frequent features. Research implications are included in section 4, and section 5 is the article's conclusion. Frequently used abbreviations in the article are listed below:

## 2 RESEARCH MATERIALS AND METHODS

This study has been performed according to PRISMA guidelines [9], a widely accepted scientific framework for reporting systematic reviews and meta-analyses. The four main PRISMA steps are identification, screening, eligibility, and inclusion. In the identification phase, researchers report the number of records identified through database searching and other sources. Duplicate records are removed in the screening phase while reporting the number of records that need to be screened and the number of records excluded. Studies should be filtered by excluding irrelevant articles through proper eligibility criteria. In the eligibility phase, the number of full-text articles assessed for eligibility and those excluded should be reported with reasons. Finally, the number of studies included in the systematic review should be specified at the inclusion [9]. The repositories used for article selection, the methods used to extract the most relevant articles, excluding criteria, and reporting the results are milestones of conducting a more sound and scientific systematic review.

The current systematic review study adopts the guidelines of the PRISMA method to conduct and report the research results. The research article identification has been done through a well-known article repository, “Web of Science” [12], which provides a consistent search interface to multiple databases of academic journals, conference proceedings, letters, and other related publications in various disciplines. The research articles are identified using a scientifically- structured search query:

Query ((“risk*” or “risk model*” or “risk assess*” or “risk equation*” or “risk predict*”) AND (“Diabetes*” or “Complication of diabetes” or “Complications of diabetes” or “comorbidities of diabetes*” or “comorbidity of diabetes*” or “diabetic*”) AND (“Statistical model*” or “Regression model” or “Cox*” or “Artificial Intelligence*” or “*model*” or “Time series analysis*” or “Machine Learning” or “Time series Forecasting”)).

The search has been filtered under publication year, document type, accessibility, and publication journal. Research papers published within the last eight years (2015-2023) were considered in this study. The latest electronic search was performed on 1^st^ July, 2023. The reviews, proceeding papers, meeting abstracts, editorial materials, book chapters, letters, and news items were filtered out from the search results due to the possible uncertainties and inconsistencies that they might bring. Furthermore, only open-access articles were selected for this study. Fifteen journals with the highest impact factor were chosen as top-ranked journals among the resulting journals from the search query. The details of the journals can be found in the appendix (Table **A1**). The resulting articles were manually selected as eligible for the study by considering their relevance to the research topic. The relevancy criteria focused on the aim of the research study, the presence of considered risk factors, and the type of features they considered. Finally, articles have been divided into four peers according to the complication type. The flow diagram of the article selection of the study is illustrated in Fig. (**1**).

The feature list for each complication has been extracted by reviewing the features used in selected studies. The selection criterion for the frequent features is their presence in at least 20% of selected articles. For example, to be selected as a frequent feature for DNeu, the feature should be present in at least 4 of the 20 selected articles (20%) in that category. The frequency of each complication has been set as the threshold value to determine the features. The extracted frequent feature set has been categorised using the USCDI standards, resulting in nine categories. Although the lifestyle features were unavailable in EHR, we included them in a separate category as lifestyle features due to their importance in some research papers. The highly frequent feature list was created by selecting the common features for all complications, with 20% or more in each complication. The frequencies of the features were calculated as percentages to visualise their utilisation in each complication. Moreover, the study's second objective describes the identified top eight features common to all four complications. Section 3.2 describes the features to provide a sound understanding of features, their usage in studies, and the significant factors about them.

## 3 RESULTS AND DISCUSSION

The findings of the study are presented and discussed in this section. The identified set of features is mentioned in the first part of the section, while the selected persistent features are described later.

### 3.1 Risk Factors for Predicting Complications of Diabetes Mellitus

**Fig. (2):**
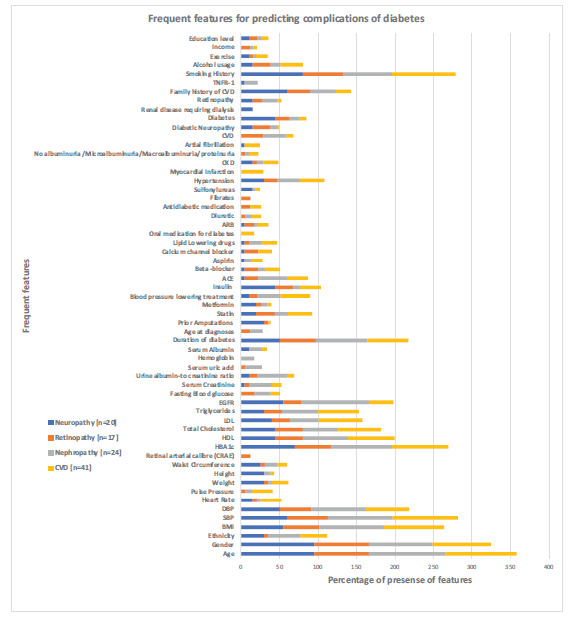
Percentages of selected frequently used features for predicting the complications of diabetes.

**Fig. (A1):**
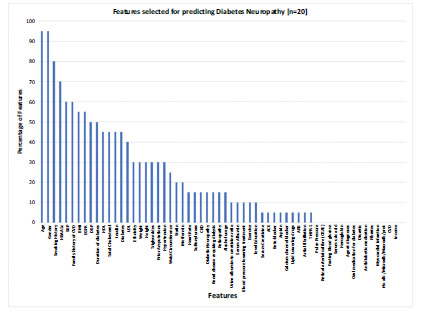
Percentage of features extracted for predicting diabetic neuropathy.

**Fig. (A2):**
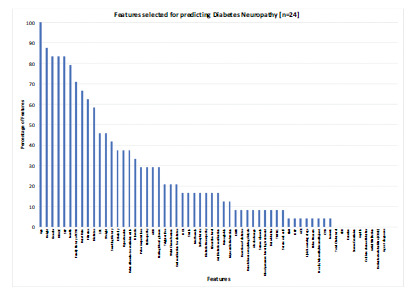
The percentage of features in diabetic nephropathy.

**Fig. (A3):**
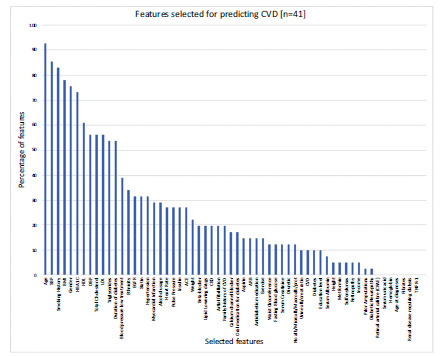
The percentages of features in CVD.

**Fig. (A4):**
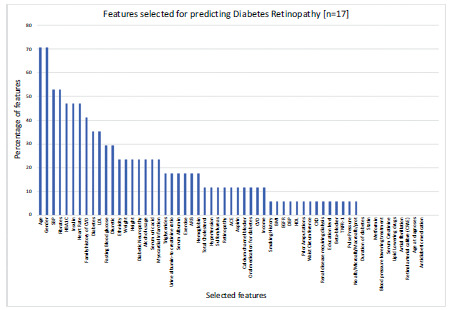
The percentages of features in diabetic retinopathy.

The selected features of complications are illustrated with their percentages in (Fig. **2**). Among the chosen attributes, age, gender, ethnicity, weight, height, BMI, smoking history, HbA1c, SBP, eGFR, DBP, HDL, LDL, total cholesterol, triglyceride, use of insulin, duration of diabetes, family history of CVD, diabetes has been recognised as the feature subset, which can be used in risk prediction of all four complications. According to the selected feature list, predicting CVD gets the most significant number of features (n=29), while DR has the most minor features (n=19). DNeu and DNeph result with 24 features. Age and gender remain the two most frequent features for DNeu and DR, while gender is the third-highest priority for CVD and DNeph. Although the absence of a percentage of a factor in one complication represents its infrequency, it does not mean the invalidity of it in predicting that complication. For example, the percentage of the feature of “urine albumin to creatinine ratio” only shows in DNeph, but it had been used for all other complications less frequently. Some features have been extracted due to their frequency in one complication, which is entirely unrelated to other complications. “Retinal arterial calibre” is a feature that has been selected due to its frequency in DR, which cannot be used anywhere else. “Renal disease requiring dialysis”, “Myocardial infraction,” and “Fibrates” are a few other features that have been selected based on one complication. Moreover, the terminology used in different articles varies hugely. Due to the requirement to extract the terms in the papers as they were, some feature terms may overlap. For example, “Metformin” and “Oral medication for diabetes” are two feature values. Furthermore, the term “Antidiabetic medication” is also included under the medications of diabetes to maintain the authenticity of the words. The resulting plots of percentages of risk factors in each complication are included in Appendix (Figs. **A1**-**A4**).

The articles used for extracting the feature sets for the complications are tabulated in Table **1**.

The table consists of columns representing the complications, while the rows represent the selected features and their sub-categorisation. The total number of studies is mentioned in each cell to emphasise the significance of the feature in each complication. The total number of studies used in each complication has been written with the complication type in the table headers. Furthermore, Table **1** can be used as a reference in finding the articles for predicting CoDM and retrieving the articles based on specific risk factors.

### 3.2 Introducing Highly Prioritised Risk Factors

The following section describes the selected attributes as the top frequent features in predicting selected complications. The identified top eight features of the study: Age, gender, BMI, eGFR, SBP, HbA1c, smoking history, and DBP are described in the following section to provide a concise description of their usage in different studies. Due to the potential importance of the top eight features in describing their nature in the literature, the highly ranked eight features are selected for the description.

#### 3.2.1 Age

Age is the number one predictor in CVD, DNeu, and DNeph prediction. Since age is a patient's primary demographic detail, the feature's availability in EHR is high. Age is also considered a categorising factor for representing the prevalence of diabetes mellitus [105]. According to the IDF, the age group 76-80 had the highest prevalence of DM (19.9%), while the age group 20-25 maintained the lowest prevalence rate (1.4%) in 2019 [105]. Age is considered a decisive risk factor in many studies [13, 28, 30, 31, 32, 62, 64, 65]. Additionally, increasing age is the most vital risk factor for clinical CVD [27, 65]. Older age is a highly useful feature for clinically significant CSME in patients with DM [5]. Furthermore, young people with T2DM show a significantly higher risk of cataract surgery than non-diabetes [33]. It has been found that the death risk rate of patients with DNeu was higher at younger ages than at older ages [14]. Furthermore, there is a high risk of DPN with increasing age [13, 15]. Moreover, the considered participants of studies showed a variety of age ranges: 41-70 [13, 15], 16-90 [16], 26-85 [7], 31-80 [66], 36-90 [17], 46-65 [18], >=66 [32], 41-81 [34], 51-74 [31], and 20-75 [30]. Thus, age has been categorised into different groups in different studies, as mentioned in Table **2**.

#### 3.2.2 Gender

Gender has been ranked as the highest prioritised feature in DR, while it remains in the top four positions in others. According to the IDF, the prevalence of DM in women (9.0%) in the age of 20-80 is slightly lower than that of men (9.6%) [105]. A retrospective cohort study based on the clinical data in England found that men were diagnosed 2.6 years earlier than women, and the CVR risk factor management was worse in women than men [67]. Considering other factors, such as standardised BMI values, gender plays a vital role in predicting CoDM. Wright *et al.* concluded that CVD risk is higher in men than women with T2DM [69]. Patients diagnosed with T2DM at a young age (18-45 years) were more frequently male than female [68]. Thus, there was a consistent association between CVD and the male sex [70]. However, the lipid profile of patients is not highly associated with gender [3]. No effect modification has been found with the sex disparity among patients with cardiovascular autonomic neuropathy [71]. Furthermore, CVD and major adverse cardiovascular events (MACE) were not associated with sex [65, 67]. Additionally, a significant association cannot be found between sex and incident DPN [13]. Braffett *et al.* concluded a weak association between sex and cardiovascular autonomic neuropathy (CAN) [3]. Moreover, the gender values of the results of studies are reported as female [1, 72-74], male [3, 4, 15, 65, 68, 75-77], and also as female and male as two categories [16, 21, 22, 67, 70, 78, 79].

#### 3.2.3 BMI

BMI is calculated as the weight (kg) of a person divided by their height squared (m^2^) [68]. BMI and waist circumference are related to obesity of a person, which are considered clinical inflammation factors for DR [35]. The prevalence of DM is higher in patients who are overweight or obese [66]. Obesity has been recognised as a risk factor for DPN [13, 15]. “Evidence showing the association of obesity and DR remains equivocal” [35]. Furthermore, a significantly high mean BMI can be seen among patients with CAN [77]. Additionally, an inverse relationship has been proven between the age at diagnosis of T2DM and BMI [80]. The BMI is eight units higher in patients diagnosed with T2DM at <41 years old than those who developed T2DM in their 91 s [80]. Thus, patients with early diagnosis (18-45 years) have high mean BMI values [68]. The baseline value of BMI is higher in women than men at the diagnosis of T2DM [67]. The high risk of CSME and ocular surgery was associated with increased BMI [5]. Although BMI is a well-established risk factor for predicting CVD among patients with T2DM, BMI or obesity did not emerge in some studies [65]. A well-established online risk assessment tool for CoDM and its extensions, such as QRISK2 and QRISK3, use BMI as a decisive risk factor for predicting CVD [6]. Furthermore, BMI values are categorised in different ways by different authors. Table **3** represents some of the categorisations of BMI values in literature.

#### 3.2.4 eGFR

eGFR is the most commonly used test in evaluating kidney functions based on the creatinine level of the blood. eGFR and albuminuria are the traditional markers for predicting CKD [41]. However, it has been recognised that eGFR decline was not significantly different from T1DM to T2DM in a mixed linear model [42]. There are two popular equations in calculating eGFR value, such as Chronic Kidney Disease Epidemiology Collaboration (CKD-EPI) [41, 43-54] and Modification of Diet in Renal Disease (MDRD) [42, 55-57]. Additionally, some studies used the Zappitelli combined equation and FAS equation [58]. Categories of eGFR are varied among researchers. Penno *et al.* divided the values of eGFR into three categories: >=91 ml min^-1^, 61-90 ml min^-1^, and <61 ml min^-1^ [55]. Furthermore, five categories of eGFR values: G1 (>=91), G2(61-90), G3a (46-60), G3b (31-45), and G4 (15-30) can be found in a study [45] (all the categories are in the unit of ml/min/1.74 m2). (<15, 15-30, 31-45, 46-60, 61-90, >=91 [33]), (<61, 61-75, 76-90, >=91 [55]), (<61,>=61 [56, 65, 104]), (<31, 31-60, 61-90, >=91 [53]) are some examples of other existing category types.

#### 3.2.5 SBP (Systolic Blood Pressure)

SBP measures the pressure of the blood exerting against the artery walls when the heart beats. SBP shows the highest frequency in predicting CVD and DR. Greater values of SBP than 141 mm Hg are more common in diabetes patients than in non-diabetes [66]. SBP values are differently categorised literature. Bragg *et al.* made three categories of SBP as <120, 120- 140, >= 141 [66], and Ku. *et al.* created four categories (<120, 120 -<131, 131- <141, >=141 [51]). Higher SBP has been selected as a significant predictor in previous studies [65, 66]. An apparent reduction of SBP (-6.31 mmHg) results from an increase of “moderate to vigorous-intensity physical activity” among the individuals who increased their physical activity for over four years of follow-ups [81]. SBP was considered a risk factor in QRISK2, a well-known algorithm for predicting the risk of developing cardiovascular disease in the next 10-year period. The upgraded version (QRISK3) of QRISK2 used the SBP and the variability of SBP as two risk factors. Although including the standard deviation of blood pressure did not improve the discrimination and calibration in predicting CVD, the SBP and its variability were independently associated with an increased risk of CVD [6]. The higher mean value of SBP has been stabilised as a risk factor in predicting any CVD [77]. Rørth *et al.* also concluded that considering SBP as a time-varying covariate did not show a disparity between diabetes and non-diabetes. High mean SBP can be seen in foot ulceration in patients with T2DM, while no significant disparity can be seen in T1DM. SBP has been recognised as the top risk factor for predicting CAN [3].

#### 3.2.6 HbA1c

HbA1c is a well-established measurement for diagnosing DM, which measures the average blood glucose amount for one to two months. A strong relationship between HbA1c and CVD has been concluded [14]. Furthermore, Andreson *et al.* concluded that “the rate of HbA1c increase affects the development of diabetic polyneuropathy over and above the effect of the HbA1c level” [15]. A study revealed a paradoxical finding of a gradual increase of HbA1c among the patients who show better baseline glycaemic control [22]. Moreover, the higher mean value of HbA1c is recognised as the greatest risk factor for predicting proliferative DR and CSME [5]. HbA1c is concluded as the strongest risk factor for the progression of DR [5]. A U-shaped relationship has been found between HbA1c and mortality, where the modest glycaemic control (HbA1c 7.1-8.0%) shows the lowest mortality risk in patients with T2DM and CHF [74]. Some researchers consider the HbA1c values as a definite factor. Elder *et al.* categorised it into three classes, <=7.0, 7.1-9.0, and >9.0, where five categories of HbA1c classes were used to separate the participants at baseline, such as <6, 6.1-7.0, 7.1-8.0, 8.1-9.0 and >9 [74]. Moreover, ((6.5-8.0%), (<6.5%), (>8.0%) [22]), ((<=7.0% or <=54 mmol/mol), (7.1- 8.0% or 55-65 mmol/mol), (>8.0% or >65 mmol/mol), (unknown) [36]) are some of the other categories.

#### 3.2.7 Smoking History

Smoking history is a significant behavioural feature frequently used in risk model creation in all four considered complications. This feature has been ranked in the top three features in CVD and DNeu. The smoking history data has been collected under various categories. Table **4** presents existing categorisation in collecting, analysing and reporting the details of smoking history in literature.

A progressive increase in CVD risk with smoking status among diabetes patients has been confirmed by Bragg *et al.* [66]. However, few researchers stated that there is no statistically significant association between the risk of DPN and smoking [13, 15]. Furthermore, smoking has not been selected in two models created by Penno *et al.* as a significant independent risk factor in predicting CKD among diabetes patients [55].

#### 3.2.8 DBP (Diastolic Blood Pressure)

DBP is the pressure in arteries when the heart rests between beats. The standard value of DBP is 81mmHg. DBP ranks higher in CVD and DNeu than in the other two complications. Elevated DBP is linked to higher rates of kidney function decline in diabetic kidney disease [41-43, 53, 54, 59]. An association between specific DBP patterns and glomerular filtration rate has been recognised among patients with DNeph [59]. Moreover, DBP is linked to early cardiac dysfunction in diabetes [70, 72, 84, 90]. DBP is also considered sex-specific in predicting occlusive vascular and mortality outcomes in diabetes [14]. Scholars found evidence of the association between diuretic use, increased DBP, and lower limb events in type 2 diabetes [23, 27], while the TODAY study confirmed that metabolic factors, race-ethnicity, and sex influence nephropathy development concerning DBP [64].

### 3.3 Discussion on Features used in Predicting CoDM

As the result of this study, we identified 60 features frequently used as predictors in CoDM. Nineteen features are recognised as common in all four considered complications, which are age, gender, ethnicity, weight, height, BMI, smoking history, HbA1c, SBP, eGFR, DBP, HDL, LDL, total cholesterol, triglyceride, use of insulin, duration of diabetes, family history of CVD, and diabetes. Although we consider the frequent features used in literature, some features may have significant importance in predicting CoDM but are not used as frequently as the other traditional features used in the literature. The genetic risk factors are an excellent example of features not commonly used to design risk prediction models but possess significant prediction power. Recently, there has been a trend in using genetic factors [94] and biomarkers [63] for predicting CoDM. Single nucleotide polymorphisms (SNP) are used vastly as a genetic factor for predicting CVD [94]. Furthermore, the biomarkers TNFR-1, TNFR-2, and KIM1 have been used in predicting diabetes nephropathy. Moreover, diagnosis and prognosis of diabetes retinopathy have been done with image processing techniques. Therefore, the features used in this complication are varied from the rest. Retinal arterial calibre (CRAE) [31, 41], arteriolar tortuosity, and fractal dimensions are commonly used in predicting DR with image processing techniques [39]. The effect of retinal venular tortuosity and fractal dimensions in predicting incident retinopathy is explored to understand their prediction capability [38]. The feature categories used terminology and how features are used vary among scholars. The usage of features in prediction models is hard to generalise since data availability, the nature of data sources, the focus of the risk model, and selected ML techniques are hugely different.

## 4 RESEARCH AND CLINICAL IMPLICATIONS

### 4.1 Research Implications

The findings of the research study have shown significant academic and clinical impact. The identified set of features of the CoDM is highly useful for researchers who predict CoDM with statistical and computerised prediction models. By systematically extracting and analysing frequently used risk factors from the existing literature, the review sheds light on the critical determinants contributing to the development and progression of complications associated with diabetes. The feature set can be used as a quick reference in feature selection and feature engineering phases in model creation. Identifying these risk factors not only advances our understanding of the multifaceted nature of diabetes complications but also provides a comprehensive framework for future research endeavours. Furthermore, academics can use the feature set to validate and prove the credibility of their feature selection not only against individuals but also against a thorough literature review, which enhances the model's acceptability. Moreover, the provided list of articles that use the identified features in each complication guides academics to extract the relevant papers for future studies effectively.

### 4.2 Clinical Implications

Clinicians, general practitioners, policymakers, and other stakeholders in the industry of healthcare can use the findings of this research to update their knowledge of the domain. The most frequently used feature set and the brief descriptions of selected features provide state-of-the-art and updated knowledge, beneficial in disease diagnosis and decision-making. Clinicians and healthcare professionals can utilise the identified risk factors as valuable tools to assess the likelihood of complications in diabetic patients, enabling personalised and targeted intervention strategies. By integrating these risk factors into clinical assessments, healthcare providers can proactively identify patients at higher risk of nephropathy, neuropathy, cardiovascular disease (CVD), and retinopathy, facilitating early intervention and more effective complication prevention. The importance of each feature in predicting the complications provides a direction for clinicians to investigate them to make informed decisions. The current study's findings can adapt to future risk prediction, disease diagnosis and prognosis models. Additionally, the overall outcome of the study provides a thorough state-of-the-art, which keeps the clinicians and other stakeholders updated with the domain knowledge.

## CONCLUSION

Disease prognosis based on ML algorithms with various risk factors has become a prominent research area due to its convenience and cost-effectiveness. Risk scoring models are frequently created to fulfil the purpose of non-invasive or minimally invasive risk prognosis. Since the model accuracy and reliability depend highly on the selected feature set, choosing the most appropriate feature set is vital prior to model designing. This systematic review focuses on extracting the most frequently used attributes for predicting the complications of diabetes mellitus through utilising the features of EHR. Due to their elevated and irreversible health effects, DNeu, DNeph, DR, and CVD are selected as severe CoDM. After searching the related articles for the recent eight years in top-ranked journals, the most common facet set that belongs to each category of complication was selected. According to the results, fifty-nine features have been chosen as frequent features for predicting the selected CoDM. Among them, age, gender, ethnicity, weight, height, BMI, smoking history, HbA1c, SBP, eGFR, DBP, HDL, LDL, total cholesterol, triglyceride, use of insulin, duration of diabetes, and family history of diabetes CVD are determined as the persistent features in the prediction of CoDM. The identified feature set provides meaningful exploration of the state-of-the-art features used in the feature selection phase of model designing at risk prediction model creation. Furthermore, future researchers can use this feature set to validate their selected feature sets against the state of the art. The extracted feature set from the study can be used for future research to identify the most frequent features in predicting each CoDM. Moreover, the results can be used as a reference to validate the feature sets that individual researchers would extract. The valuable implications of this study are the effectiveness of adapting the identified feature set in future studies and the capability of benchmarking the feature set of individual designs to an extracted feature set resulting from a properly performed systematic review. Due to various features with different scaling systems, categories, and uses in the studies, it is worthwhile to make a platform for discussing the similarities and variations of used features in various studies. The given introduction of the selected features provides a concise description of the usage of the feature in past studies, which is vital in feature selection and feature engineering. The outcomes of this systematic review are helpful for academics and stakeholders in the healthcare sector to understand the domain for making informed decisions. In summary, the systematic review has profound implications for the research and clinical domains. By systematically uncovering frequently utilised risk factors, the review not only advances our understanding of diabetes complications but also empowers researchers and clinicians alike to collaboratively shape the future of diabetes management, prevention, and patient care.

## AUTHORS' CONTRIBUTIONS

M. A. and YCW wrote the main manuscript text, CCL, MM, and WYC did the editorial works, CCL provided ideas from the clinical aspects, and YCW did the final proofreading and submissions.

## Figures and Tables

**Fig. (1):**
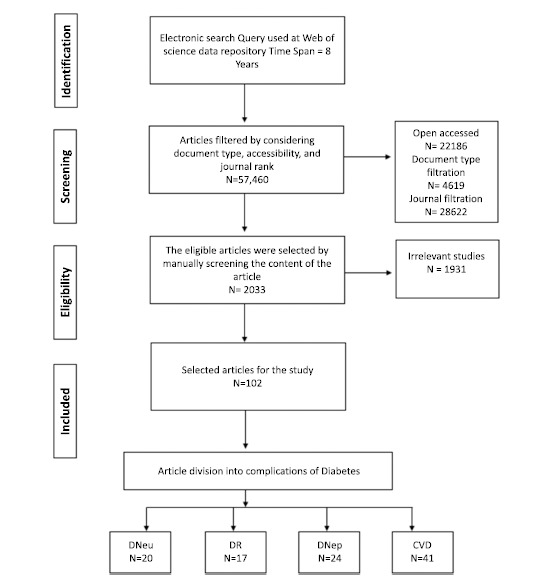
Flow diagram of the article selection.

**Table 1:** Articles used to extract the frequent features of each complication. The articles cited in the table are included in the square braces, while the number of articles enclosed in braces.

**No.**	**Attribute Category**	**Attributes**	**Neuropathy (n=20)**	**Retinopathy (n=17)**	**Nephropathy (n=25)**	**CVD (n=42)**
1	Demographic	Age	[2, 3, 13-29] (n=19)	[5, 30-40] (n=12)	[41-64] (n=25)	[1,4,6,65-99] (n=38)
2		Gender	[2, 3, 13-29] (n=19)	[5, 30-38, 40, 100] (n=12)	[41-51, 54-57, 60-64] (n=20)	[1, 4, 65, 67, 68, 70-78, 80, 82, 84-93, 95, 98, 99, 101, 102] (n=31)
3		Ethnicity	[18, 19, 20, 22, 24, 29] (n=6)	[40] (n=1)	[41, 45, 46, 50, 51, 57, 58, 62-64] (n=10)	[6,66-69, 73, 80, 82, 85, 87, 88, 92, 93, 99] (n=14)
4	Vital Signs	BMI	[3, 13-15, 17, 18, 22-24, 27, 29] (n=11)	[5, 30, 33-36, 38, 40] (n=8)	[41-47, 49-54, 56-58, 60, 62-64] (n=20)	[1, 4, 6, 65-68, 70-81, 84-89, 91-93, 96-98, 103] (n=32)
5		SBP	[3, 13-16, 18, 21, 23, 24, 27-29] (n=12)	[5, 30, 32, 33, 35, 36, 38, 40,100] (n=9)	[41-49, 52, 53, 55-60, 62-64] (n=20)	[1, 4, 6, 51,65-73, 77, 78, 80-82, 84-98, 102,104] (n=35)
6		DBP	[3, 13, 15, 16, 18, 23, 24, 27-29] (n=10)	[5, 30, 32, 33, 35, 40, 100] (n=7)	[41-45, 47, 49, 52, 53, 55-60, 62, 63] (n=17)	[1, 4, 65, 67-73, 77, 78, 80, 82, 84-87, 89, 92, 97, 98, 103] (n=23)
7		Heart Rate	[6, 28, 66] (n=3)	[17] (n=1)	[52] (n=1)	[4, 65, 72, 73, 77, 82-84, 89, 90, 104] (n=11)
8		Pulse Pressure	(n=0)	[17] (n=1)	[52, 56] (n=2)	[4, 65, 67, 71, 72, 84, 88, 94-96, 104] (n=11)
9		Weight	[3, 13-15, 24, 28] (n=6)	[17] (n=1)	[52] (n=1)	[4, 65, 71, 72, 79, 81, 82, 84, 91] (n=9)
10		Height	[3, 13-15, 17, 28] (n=6)	(n=0)	[47, 62] (n=2)	[81, 91] (n=2)
11		Waist Circumference	[13-15, 17, 21] (n=5)	[79] (n=1)	[43, 45, 53, 55] (n=4)	[70, 71, 81, 83, 86] (n=5)
12		Retinal arterial calibre (CRAE)	(n=0)	[31, 41] (n=2)	(n=0)	(n=0)
13	Lab Orders/ Values	HbA1c	[2, 3, 13,15-17, 21-25, 27-29] (n=14)	[5, 30, 32-34, 36, 38, 100] (n=8)	[42-49, 52-60, 62, 63] (n=19)	[1, 4, 65, 67-78, 80, 81, 84, 85, 88-91, 94-98, 102, 103] (n=30)
14		HDL	[3, 13, 15, 16, 18, 23, 24, 27, 29] (n=9)	[5, 33, 34, 38, 40, 100] (n=6)	[41, 43, 45, 47-49, 52-55, 57, 58, 60, 62] (n=14)	[4, 65, 67, 70-73, 75-78, 80, 81, 83-88, 92, 93, 97, 98, 102, 103] (n=25)
15		Total Cholesterol	[3, 13-18, 23, 24] (n=9)	[5, 33, 34, 38, 40, 100] (n=6)	[41, 43-45, 47, 52, 54-56, 60, 62] (n=11)	[1, 4, 6, 65, 68, 70-73, 75-77, 80, 84-87, 91-93, 98, 99, 104] (n=23)
16		LDL	[3, 13, 15, 16, 23, 24, 27, 29] (n=8)	[5, 30, 32, 40] (n=4)	[41, 47-49, 52, 53, 55, 57, 62] (n=9)	[1, 4, 65, 67, 70-73, 75-78, 80, 83-86, 90, 92, 94-96, 98] (n=23)
17		Triglycerides	[3, 13, 15, 17, 23, 24] (n=6)	[5, 33, 34, 40] (n=4)	[41, 43, 45, 47, 48, 52, 53, 55, 58, 62, 64] (n=11)	[4, 65, 70-73, 75-78, 80, 81, 83, 84, 86, 90, 92, 94-96, 98, 104] (n=22)
18		EGFR	[2, 3, 17, 18, 21-24, 26, 27, 29] (n=11)	[5, 30, 33, 100] (n=4)	[41-43, 45-51, 53-60, 62-64] (n=21)	[1, 65, 68, 71, 73-75, 80, 85, 91, 95, 103, 104] (n=13)
19		Fasting Blood glucose	(n=0)	[33, 34, 44] (n=3)	[44, 45, 55, 56, 60] (n=5)	[45, 76, 83, 86, 98] (n=5)		
20		Serum Creatinine	[16] (n=1)	[41] (n=1)	[45, 48, 51, 55, 56, 58, 60] (n=7)	[75, 82, 92, 93, 98] (n=5)		
21		Urine albumin-to-creatinine ratio	[57, 59] (n=2)	[33, 38] (n=2)	[41, 43, 46, 47, 56-58, 62, 63] (n=9)	[45, 78, 88, 98] (n=4)		
22		Serum uric acid	(n=0)	[32] (n=1)	[45, 49, 55, 60, 64] (n=5)	(n=0)		
23		Haemoglobin	(n=0)	(n=0)	[44, 50, 51, 60] (n=4)	(n=0)		
24		Serum Albumin	[15, 72] (n=2)	(n=0)	[50, 53, 54, 62] (n=4)	[45, 71, 91] (n=3)		
25	Diagnoses	Duration of diabetes	[2, 3, 15, 21-25, 28, 29] (n=10)	[5, 30, 33-35, 38, 100, 103] (n=8)	[42, 44-49, 51-55, 57-59, 62] (n=16)	[1, 4, 65, 67, 71-75, 77, 78, 84, 85, 90,91, 93-97, 102, 103] (n=22)		
26		Age at diagnoses	(n=0)	[79, 100] (n=2)	[45, 47, 55, 59] (n=4)	(n=0)		
27		Prior Amputations	[20-25] (n=6)	[79] (n=1)	(n=0)	[80] (n=1)		
28	Medication	Statin	[13, 15, 19, 23] (n=4)	[30, 32, 33, 36] (n=4)	[43, 49, 53, 61] (n=4)	[66, 68, 73-76,80, 85, 87-89, 92, 103] (n=13)		
29		Metformin	[15, 22, 29, 57] (n=4)	[32] (n=1)	[53, 55] (n=2)	[45, 89] (n=2)		
30		Blood pressure lowering treatment	[29, 59] (n=2)	[36, 41] (n=2)	[42, 43, 47, 49, 54, 61, 62] (n=7)	[1, 66, 67, 68, 73, 78-81, 83, 85, 87, 88, 92, 99, 102] (n=16)		
31		Insulin	[2, 3, 15, 21-25, 29] (n=9)	[33, 38, 40, 79] (n=4)	[53, 55] (n=2)	[65, 66, 68, 72-76, 78, 89, 103] (n=11)		
32		ACE	[66] (n=1)	[5, 32, 33] (n=3)	[41, 42, 48, 53, 54, 58, 59, 61, 63] (n=9)	[65, 73-76, 78, 85, 89, 90, 94, 96] (n=11)		
33		Beta-blocker	[66] (n=1)	[5, 32, 33] (n=3)	[61, 62] (n=2)	[65, 73-76, 78, 82, 89] (n=8)		
34		Aspirin	[15] (n=1)	(n=0)	[53, 57] (n=2)	[66, 73-76, 89] (n=6)		
35		Calcium channel blocker	[66] (n=1)	[5, 32, 33] (n=3)	(n=0)	[65, 73-75, 78, 82, 89] (n=7)		
36		Lipid Lowering drugs	[17] (n=1)	[41] (n=1)	[43, 50, 55, 62] (n=4)	[1, 65, 67, 73, 78, 79, 81, 99] (n=8)		
37		Oral medication for diabetes	(n=0)	(n=0)	(n=0)	[66, 69, 73, 75, 76, 78, 89] (n=7)		
38		ARB	[66] (n=1)	[32, 33] (n=2)	[63] (n=1)	[65, 73-76, 78] (n=6)		
39	Medication	Diuretic	(n=0)	[32] (n=1)	[49, 61] (n=2)	[73, 75, 78, 82, 89] (n=5)		
40		Antidiabetic medication	(n=0)	[36, 38] (n=2)	(n=0)	[76, 79, 81, 87, 96, 103] (n=6)		
41		Fibrates	(n=0)	[33, 36] (n=2)	(n=0)	(n=0)		
42		Sulfonylureas	[22,57,59] (n=3)	(n=0)	[53] (n=1)	[44, 73] (n=2)		
43	Problem List	Hypertension	[3, 16, 17, 22, 23, 29] (n=6)	[5, 31, 35] (n=3)	[51-53, 55, 57, 61, 63] (n=7)	[4, 6, 65, 68, 70, 75, 76, 78, 82, 84, 89, 91, 103] (n=13)
44		Myocardial Infarction	(n=0)	(n=0)	(n=0)	[4, 65, 70, 74, 80, 82, 84, 89, 90, 94-96] (n=12)
45		CKD	[22, 57, 69] (n=3)	[32] (n=1)	[42,45] (n=2)	[6, 69, 78, 80, 84, 88, 89, 96] (n=8)
46		No albuminuria /Microalbuminuria/Microalbuminuria/proteinuria	[27-29] (n=3)	[17] (n=1)	[51] (n=1)	[1, 67, 68, 75, 88] (n=5)
47		Atrial fibrillation	[27] (n=1)	(n=0)	(n=0)	[6, 12, 80, 82, 88, 91, 99, 102] (n=8)
48		CVD	(n=0)	[31-33, 36, 79] (n=5)	[41, 45, 50, 53, 55, 57, 61] (n=7)	[73, 80, 82, 89] (n=4)
49		Diabetic Neuropathy	[17, 21, 25] (n=3)	[32, 33, 35, 36] (n=4)	[45,55] (n=2)	[67] (n=1)
50		Diabetes	[3, 14, 17, 18, 20, 22, 25, 26, 28] (n=9)	[31, 35, 100] (n=3)	[43,46,50] (n=3)	[4, 6, 65, 66] (n=4)
51		Renal disease requiring dialysis	[12, 22, 69] (n=3)	(n=0)	(n=0)	(n=0)
52		Retinopathy	[12, 22, 57] (n=3)	[39, 40] (n=2)	[41,47,51,54,55] (n=5)	[67, 88] (n=2)
53	Family History	Family history of CVD	[2, 3, 13, 16-18, 20, 22, 23-26] (n=12)	[31-33, 35, 36] (n=5)	[41, 45, 50, 53, 55, 57, 61, 62] (n=8)	[6, 67, 69, 73, 76, 80, 83, 89] (n=8)
54	Bio-sample Data	TNFR-1	[17] (n=1)	(n=0)	[41,44,62,63] (n=4)	(n=0)
55	Lifestyle Features	Smoking History	[3, 13-18, 20-26, 27, 29] (n=16)	[5, 30, 31, 33-36, 38, 40] (n=9)	[42-44, 47-49, 52-57, 59, 62, 63] (n=15)	[1, 4, 6, 65, 66, 68-71, 74-79, 80-84, 85-93, 95, 96, 98, 102, 103] (n=34)
56		Alcohol usage	[13, 15, 17] (n=3)	[5, 31, 33, 36] (n=4)	[47, 52, 62] (n=3)	[4, 65, 66,76,78, 79, 81, 84, 86, 89, 93, 103] (n=12)
57		Exercise	[2, 76] (n=2)	[17] (n=1)	[62] (n=1)	[65, 66, 80, 81, 86] (n=6)
58		Income	(n=0)	[32, 35] (n=2)	[61] (n=1)	[1,80] (n=2)
59		Education level	[12, 55] (n=2)	[31, 79] (n=2)	[51] (n=1)	[1, 66, 68, 80] (n=4)

**Table 2:** Summarising the use of different age groups in literature.

Study No	Age Groups
[7]	26-85 with 5-year age groups
[105]	20-25, 26-30, 31-35, 36-40, 41-45, 46-50, 51-55, 56-60, 61-65, 66-70, 71-75, 76-80
[33]	46-55, 56-65, 66-75, 75-85, >=86
[14]	<=57, 58-65, 66-71, >71, 36-60, 61-70, 71-90
[16]	<41, 41-50, 51-60, 61-70, >71
[66]	31-60, 61-70, 71-80
[67]	<51, 51-61, 61-71, >=71
[68]	18-45, 46-60, 61-75, >=76
[19,20]	<26, 26-50, 51-65, 66-75, >=76
[65,72]	13 < age < 39
[64]	18-36, 36-51,51-66, >=66
[40]	<51, 51-60, 61-70, 71-80, >=81

**Table 3:** BMI categories used in the literature.

Study No.	BMI Values
[14]	<24.51, 24.5-27.76, >=27.76
[66]	<23.0, 23.0 – 26.0, >= 26.0
[68]	<31, >31
[36]	< 26, 26–30, >=31, unknown

**Note:** Some researchers recorded the BMI values of men and women separately [3-6, 65].

**Table 4:** Smoking categories used in the literature.

Study No.	Smoking Categories
[6]	Non, former, light (1-9/day), moderate (10-19/day), heavy (≥ 20/day)
[67]	Current, ex-smoker, never, unknown
[21, 65, 77, 78]	Yes, No
[13, 17, 47, 66, 81]	Non, former, current
[15, 56, 59]	Former and current
[74]	Ever smoked
[4, 30, 35, 42, 68, 70, 71, 80, 82-84].	Active smoker

## Data Availability

The used research papers are included in the reference section. The extracted results are included in the article.

## LIST OF ABBREVIATIONS

BMI: Body Mass Index
ARB: Angiotensin Receptor Blockers
SBP: Systolic Blood Pressure
CKD: Chronic Kidney Disease
DBP: Diastolic Blood Pressure
TNFR-1: Tumour Necrosis Factor Receptor-1
HbA1c: Haemoglobin A1c
CSME: Clinically Significant Macular Edema
HDL: High-density Lipoprotein
T1DM: Type 1 Diabetes Mellitus
LDL: Low-density Lipoprotein
T2DM: Type 2 Diabetes Mellitus
eGFR: Estimated Glomerular Filtration Rate
DPN: Diabetic Peripheral Neuropathy
ACE: Angiotensin-converting-enzyme
AER: Albumin excretion rate

## STANDARDS OF REPORTING

PRISMA guidelines and methodology were followed.

PRISMA checklist is available on the publisher’s website.

## SUPPLEMENTARY MATERIAL

PRISMA checklist is available as supplementary material on the publisher’s website along with the published article. Supplementary material is available on the publisher’s website along with the published article.

## APPENDIX

**Table A1:** Selected journals and their importance.

**Journal Name**	**Impact Factor**
BMC Medicine	6.782
BMJ British Medical Journal	30.313
Circulation	2.5
Diabetes	7.720
Diabetes Care	16.019
Diabetologia	7.518
Environment International	7.577
European Heart Journal	22.673
European Journal of Heart Failure	11.627
International Journal of Epidemiology	7.707
Journal of the American College of cardiology	20.589
Journal of the American Society of Nephrology	9.274
Lancet Diabetes Endocrinology	25.340
Neurology	8.770
Plos Medicine	10.500
